# Oxidative Stress Regulated Iron Regulatory Protein IRP2 Through FBXL5-Mediated Ubiquitination-Proteasome Way in SH-SY5Y Cells

**DOI:** 10.3389/fnins.2019.00020

**Published:** 2019-01-29

**Authors:** Qian Jiao, Xixun Du, Jie Wei, Yong Li, Hong Jiang

**Affiliations:** Department of Physiology, Shandong Provincial Key Laboratory of Pathogenesis and Prevention of Neurological Disorders and State Key Disciplines: Physiology, School of Basic Medicine, Qingdao University, Qingdao, China

**Keywords:** oxidative stress, IRP2, FBXL5, iron metabolism, ubiquitination

## Abstract

Iron regulatory protein 2 (IRP2) plays a key role in the cellular iron homeostasis and could be regulated by a variety of factors, such as oxidative stress, hypoxia and iron, etc. IRP2 depletion results in neurodegenerative movement disorder with the loss of neurons and accumulations of iron. Since oxidative stress extensively exists in several neurodegenerative diseases where iron accumulation also exists, it is important to clarify the mechanisms underlying the effects of oxidative stress on IRP2 expression and its consequence. 200 and 300 μM H_2_O_2_ could result in the reduced cell viability in SH-SY5Y cells. The intracellular levels of reactive oxygen species (ROS) were increased by 52.2 and 87.3% with 200 and 300 μM H_2_O_2_ treatments, respectively. The decreased levels of mitochondrial transmembrane potential (ΔΨm) were only observed in 300 μM H_2_O_2_-treated group. The protein levels of IRP2, but not for its mRNA levels, were observed decreased in both groups, which resulted in the lower TfR1 expression and decreased iron uptake in these cells. Pretreatment with MG132, the decreased IRP2 levels caused by H_2_O_2_ treatment could be antagonized. The protein levels of F box and leucine-rich repeat protein 5 (FBXL5), the only E3 ligase of IRP2, were observed decreased accordingly. When knockdown the intracellular FBXL5 levels by si-FBXL5, the protein levels of IRP2 were found increased with H_2_O_2_ treatment. Our results suggest that FBXL5 is involved in the degradation of IRP2 under oxidative stress in dopaminergic-like neuroblastoma cells, which implies that its role in the neuronal regulation of IRP2 in neurodegenerative diseases.

## Introduction

Oxidative stress occurs when the accumulated production of reactive oxygen species (ROS) and the decrease of antioxidant activity. It is thought to be one of common underlying mechanisms that lead to cellular dysfunction in neurodegenerative disease (Pong, 2003; Dias et al., 2013). As such, the substantia nigra (SN) exhibits increased levels of oxidized lipids, damaged proteins, and DNA in Parkinson’s disease (PD) (Hwang, 2013). Moreover, iron levels in the SN have been reported to be elevated in patients with PD (Sofic et al., 1988; Dexter et al., 1989) and PD animal models (Wang et al., 2004; Jiang et al., 2010; You et al., 2015). Markers of oxidative stress also increase in both Alzheimer’s disease (AD) patients models brain areas in which amyloid β (Aβ) is abundant (Butterfield et al., 2001). Aβ plaques can efficiently generate ROS and accelerate iron accumulation (Smith et al., 2007; van Duijn et al., 2017). Thus, the oxidative stress may play an important role in inducing iron metabolic disorder in neurodegeneration associated with several brain pathologies.

Iron homeostasis is regulated by coordination proteins that are responsible for iron uptake, storage, exports, and utilization in the cellular (Hentze et al., 2010). Iron regulatory protein(IRPs), including IRP1 and IRP2, are RNA-binding proteins that interact with RNA stem loops known as iron responsive element (IRE) to regulate the translation and stability of mRNAs that encode proteins required for iron homeostasis, including ferritin, ferroportin (Fpn), transferrin receptor (TfR) and divalent metal transporter 1 (DMT1) (Rouault, 2006; Muckenthaler et al., 2008). It has been reported that a syndrome of progressive neurodegenerative disease and anemia develops with iron deposits in white matter and a loss of Purkinje cells in IRP2^-/-^ mice, whereas IRP1^-/-^ mice develop polycythemia and pulmonary hypertension, indicating a prominent role of IRP2 in controlling neuronal iron metabolism (Meyron-Holtz et al., 2004; Wilkinson and Pantopoulos, 2014). But some studies considered that IRP2 deficiency without symptomatic neurodegeneration in the mouse (Galy et al., 2006). Our previous study showed that IRP2 expression is decreased in 1-Methyl-4-phenylpyridinium [MPP(+)]-induced cellular model of PD (Zhang et al., 2009), whereas IRP2 expression is increased in 6-hydroxydopamine (6- OHDA)-treated PD model (Jiang et al., 2010). In AD patients, IRP2 immunoreactivity is present in the intracellular neurofibrillary, including neurofibrillary tangles and neuropil threads, which is striking differences from the control brain (Smith et al., 1998). These suggest that IRP2 expression disruption exists in neurodegenerative diseases where oxidative stress extensively occurs. In peripheral system, such as liver and kidney, IRP2 is rapidly degraded through ubiquitin-proteasomal system in high iron and high oxygen pressure conditions (Kuhn, 2015). Additionally, hypoxia and low iron level can prevent IRP2 degradation. However, in nerve system, the underlying mechanisms of oxidative stress regulate IRP2 expression and the homeostasis of iron is not fully understood.

Iron regulatory protein 2 has unique 73–amino acid that is different from IRP1 (Iwai et al., 1995). Deletion of this sequence eliminates the rapid turnover of IRP2, and does not show a sensitivity to F box and leucine-rich repeat protein 5 (FBXL5) (Iwai et al., 1995; Salahudeen et al., 2009). FBXL5, an iron- and oxygen-regulated SCF-type ubiquitin ligase (E3), has been shown to contribute to iron-dependent degradation of IRP2 in liver or human embryonic kidney cells (Salahudeen et al., 2009; Vashisht et al., 2009; Moroishi et al., 2011). Here, we used SH-SY5Y cells, a dopaminergic-like neuroblastoma cell, to investigate the regulation mechanism of IRP2 under oxidative stress condition in nerve system. We found that oxidative stress induced by H_2_O_2_ reduced IRP2 protein levels through ubiquitination-proteasome way. Our results also revealed that E3 FBXL5 plays a pivotal role in the regulation of IRP2 protein levels under oxidative stress condition in nerve system.

## Materials and Methods

### Cell Culture and Treatment

The SH-SY5Y cell line was purchased from the Cell Bank of the Shanghai Institute of Cell Biology and Biochemistry, Chinese Academy of Sciences (Shanghai, China). SH-SY5Y cells were cultured in DMEM/F12 (Dulbecco’s modified Eagle medium and Ham’s F12, 1:1, pH 7.4) with 10% fetal bovine serum (FBS), 100 U/mL of penicillin and 100 U/mL of streptomycin (all from Invitrogen, United States) at 37°C in a humidified atmosphere containing 5% CO_2._ For experiments, cells were seeded in 6 well plates with 1 × 10^5^ cells/mL and grown to 80–90% confluency before they were treated with H_2_O_2_ (20, 30, 50, 200, 300, 500, and 1000 μmol/L) for 24 h. After 24 h, the medium was replaced with medium containing H_2_O_2_, and cells were treated for another 24 h and then harvested for experiments.

### Transfection of shRNA

The FBXL5 short hairpin RNA (shRNA) and the scramble shRNA control were pre-designed and purchased from Genechem (Shanghai, CN). SH-SY5Y were transfected using 1-μg plasmid and 2.5-μL Lipofectamine 2000 reagent (Invitrogen, United States) in 800-μL transfection medium per well in 12 well plate for 24 h optimal transfection and then used for the following experiments.

### Cell Viability

The cell viability was detected by the conventional 3-(4,5-dimethylthiazol-2-yl) -2, 5-diphenyltetrazolium bromide (MTT) (Beyotime, CN) assay. The MTT assay is a colorimetric assay for measuring the activity of cellular enzymes that reduce the tetrazolium dye. Cells were incubated in MTT (5 mg/mL) for 3–4 h. 100 μL DMSO was added to each well after medium was removed. The formazan dye crystals were solubilized for 10 min, and absorbance was measured at 494 and 630 nm with a spectrophotometer (Molecular Device, M5, United States).

### Measurement of the Mitochondrial Transmembrane Potential (ΔΨm) and the Production of Intracellular Reactive Oxygen Species (ROS)

Changes in the levels of intracellular ROS and ΔΨm with H_2_O_2_ (200 and 300 μmol/L) treatment of SH-SY5Y were measured using flow cytometry with Rhodamine 123 (Sigma, United States) or carboxy-H2DCFDA dye (Invitrogen, United Kingdom) as previously described (Jiang et al., 2010). After cells were washed with HEPES buffered saline (HBS, 10 mM of HEPES, 150 mM of NaCl, pH 7.4) three times, carboxy-H2DCFDA (5 μM) was added and incubated for 30 min at 37°C. Cells were washed with HBS two times, followed by centrifugation at 800 rpm for 5 min, and re-suspended in 1 mL HBS. Intracellular ROS generation was also assayed by Rhodamine 123 (5 μg/mL). Fluorescent intensity was recorded at 488 nm excitation and 525 nm emission wavelengths (Fluorescence 1, FL1). Results were demonstrated as FL1-H (Fluorescence 1-Histogram), setting the gated region M1 and M2 as a marker to observe the changing levels of fluorescence intensity using Cellquest Software.

### Calcein Loading of Cells and Ferrous Iron Influx Assay

Ferrous iron influx were detected as previously described (Zhang et al., 2009; Du et al., 2016). Calcein-AM (Molecular Probes, United States) is a membrane-permeative, and forms fluorescent calcein by cytoplasmic esterases upon intracellular cleavage. This reaction is pH independent and can be quenched rapidly by divalent metals and reversed easily by chelators. The cells were planted in glass coverslips and incubated with calcein-AM (0.5 μM) in HBS at 37°C for 30 min. The excess calcein on the cell surface was washed for three times with HBS. Calcein fluorescence was recorded at an excitation wavelength of 488 nm and an emission wavelength of 525 nm and the fluorescence intensity was measured every 3 min for 30 min with continuous perfusion of 100 μM of ferrous iron (ferrous sulfate in an ascorbic acid solution, 1:44 molar ratio; prepared immediately before the experiments). Ascorbic acid maintained the reduced status of ferrous iron, in addition, ascorbate acted as a chelator to maintain the iron in solution. The mean fluorescence signal of 25–30 single cells in four separate fields was monitored at 200× magnification and processed with Fluoview 5.0 software.

### Quantitative Real-Time PCR

SH-SY5Y cells were collected for the investigation of FBXL5 and IRP2 mRNA expression by quantitative real-time PCR (qRT-PCR). Total RNA was extracted by using the Trizol reagent (Invitrogen) in accordance with manufacturer’s protocol and was quantified by spectrophotometry (Bio-Rad, United States). RNAs were reversely transcribed to cDNA by a reverse transcriptase kit (RevertAid First Strand Cdna Synthesis Kit, Thermo, United States). Relative abundance of each mRNA was quantified by qRT-PCR using specific primers and the SYBR Premix Ex TaqII (TaKaRa, CN). Primers for rat FBXL5 (forward 5′- TTAACTAACAAGGGCATTGGAGAAG -3′; reverse 5′- TCAGCCAAATCTTCAGCATCTAAC -3′), IRP2 (forward 5′-CGCCTTTGAGTACCTTATTGAAACA-3′; reverse 5′-CGTACAGCAGCTTCCA ACAAGA-3′) and GAPDH (forward 5′-GCACCGTCAAGGCTGAGAAC-3′; reverse 5′-TGGTGAAGACGCCAGTGGA-3′) were synthesized by TaKaRa. QRT-PCR reactions were carried out by using Real-Time PCR Detection System (Eppendorf,GER). Data were analyzed by the 2^ΔΔ^CT method, and GAPDH was taken as an internal control.

### Western Blot Analysis

The SH-SY5Y cells were washed with ice-cold PBS and lysed in lysis buffer (Cwbio, CN) containing protease inhibitors cocktail (Rhoche, GER) for 30 min. The lysates were centrifuged at 12,000 × g for 10 min, and the protein concentration of the supernatants was determined with a Pierce^TM^ BCA Protein Assay Kit (Thermo, United States). A total of 20 μg of protein was electrophoresed on 8% SDS polyacrylamide gels and transferred onto PVDF membranes (300 mA, 90 min). After blocking with 10% non-fat milk for 1 h at room temperature, the membranes were incubated overnight at 4°C with rabbit anti- FBXL5 (1:1000, Abcam, United Kingdom), rabbit anti-IRP2 (1:1000, Abcam, United Kingdom), rabbit anti-TfR1 (1:1000, Abcam, United Kingdom ) and rabbit anti-β -actin monoclonal antibody (1:10000, BIOS, CN). The cross-reactivity was visualized using ECL Kit (Millopore, United States) and analyzed through scanning densitometry with a UVP image system.

### Statistical Analysis

Results are presented as means ± SEM. One-way analysis of variance (ANOVA) followed by the Tukey’s Multiple Comparison Test was used to compare difference between means in more than two groups. The iron uptake experiment was carried out using two-way ANOVA followed by the Student-Newman-Keuls test. A probability of *P* < 0.05 was taken to indicate statistical significance.

## Results

### The Oxidative Effects of H_2_O_2_ on SH-SY5Y Cells

**FIGURE 1:**
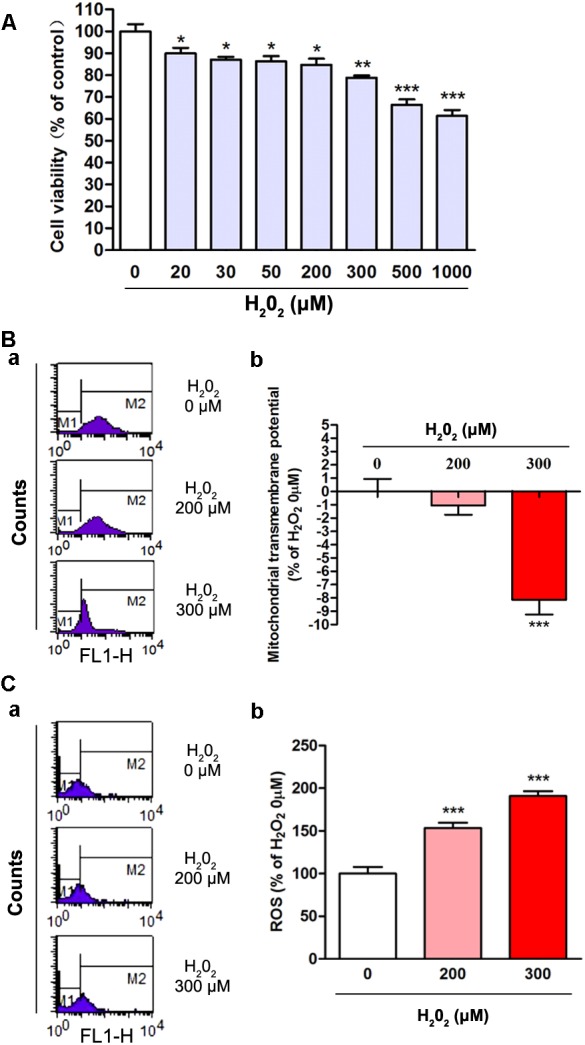
The oxidative effects of different concentrations of H_2_O_2_ on SH-SY5Y cells. **(A)** Cell viability was changed with the increased concentration of H_2_O_2_ treatment. Cell viability of the 0 μM H_2_O_2_ group was set to 100%. Data were presented as mean ± SEM of 6 independent experiments. ^∗^*P* < 0.05, ^∗∗^*P* < 0.01, and ^∗∗∗^*P* < 0.001 compared with 0 μM H_2_O_2_ group. **(B)** Fluorometric assay on ΔΨm in different groups **(a)** and statistical analysis **(b)**. ΔΨm was decreased with the increased concentration of H_2_O_2_. Fluorescence values of the 0 μM H_2_O_2_ was set to 0. Data were presented as mean ± SEM of 6 independent experiments. ^∗∗∗^*P* < 0.001 compared with H_2_O_2_ 0 μM group. **(C)** Fluorometric assay on ROS levels in different groups **(a)** and statistical analysis **(b)**. Fluorescence values of the 0 μM H_2_O_2_ group was set to 100%. Data were presented as mean ± SEM of 4 independent experiments. ^∗∗∗^*P* < 0.001 compared with 0 μM H_2_O_2_ group.

To elucidate the oxidative effects of H_2_O_2_, we assessed cell viability, ΔΨm and ROS in the present study. SH-SY5Y cells were treated with different concentrations of H_2_O_2_ for 24 h and then MTT method was used to detect the cell viability. The results showed that the cell viability was decreased with the increased concentration of H_2_O_2_ (Figure 1A). In comparing with the 0 μM H_2_O_2_ group, the cell viability in 20, 30, 50, 200, 300, 500 and 1000 μM H_2_O_2_ group was decreased by 10.2, 12.9, 13.7, 15.3, 21.2, 33.6, and 38.6%, respectively, which displayed significant differences (Figure 1A, *P* < 0.05). The 200 and 300 μM effective concentration of H_2_O_2_ were applied for the following studies. Oxidative stress induced ΔΨm reduction and excessive generation of ROS which contribute to DNA or RNA damage. We found that the ΔΨm in cells was decreased significantly in 300 μM H_2_O_2_ treatment group (Figure 1B, *P* < 0.001). The intracellular ROS levels were increased by 52.2 and 87.3%, respectively, and the difference was statistically significant (*P* < 0.001) when compared with the 0 μM H_2_O_2_ group (Figure 1C). Thus, these observations indicated that appropriate concentrations of H_2_O_2_ could induce oxidative stress in SH-SY5Y cells.

### H_2_O_2_ Induced a Reduction in IRP2 Expression and Ferrous Iron Uptake

**FIGURE 2:**
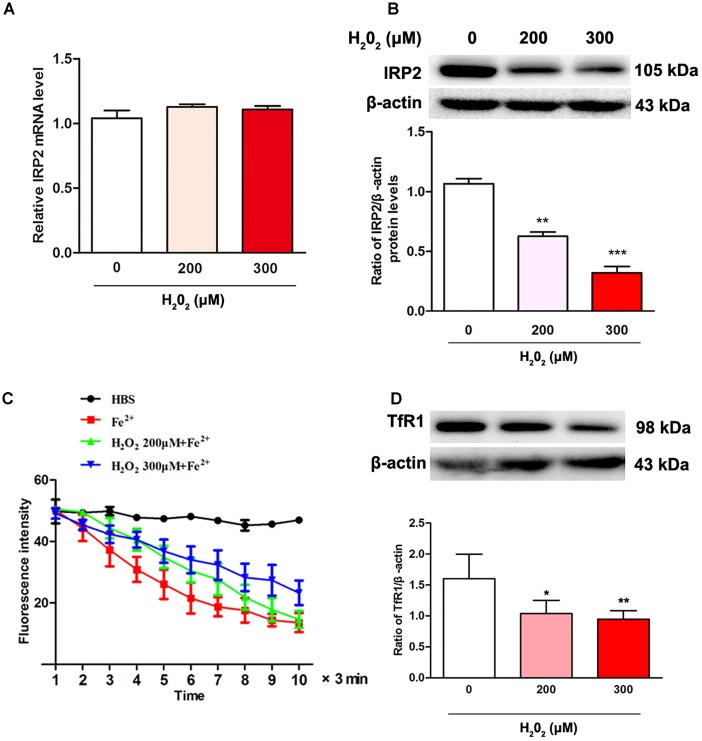
H_2_O_2_ induced the changes in IRP2 levels and ferrous iron uptake in SH-SY5Y cells. **(A)** IRP2 mRNA levels were changed with H_2_O_2_ treatment. One-way ANOVA, data were presented as mean ± SEM of 3 independent experiments. **(B)** IRP2 protein levels were changed with H_2_O_2_ treatment. One-way ANOVA, data were presented as mean ± SEM of 3 independent experiments.^∗^*P* < 0.05, ^∗∗^*P* < 0.01, and ^∗∗∗^*P* < 0.001 compared with H_2_O_2_ 0 μM group. **(C)** Calcein-indicated ferrous iron (FeSO_4_) influx in SH-SY5Y cells. The fluorescence intensity of SH-SY5Y cells in 200 μM group and 300 μM group were significantly higher than that in 0 μM H_2_O_2_ group. Two-way ANOVA, ^∗^*P* < 0.05. The mean fluorescence intensity of 35 separate cells from 4 separate fields at each time point was presented as mean ± SEM of 6 independent experiments. **(D)** TfR1 protein levels were changed with H_2_O_2_ treatment. One-way ANOVA, data were presented as mean ± SEM of 3 independent experiments.^∗^*P* < 0.05 and ^∗∗^*P* < 0.01compared with H_2_O_2_ 0 μM group.

To test the relationship of IRP2 expression and oxidative stress induced by H_2_O_2_, we examined the expression of IRP2 in mRNA and protein levels. After SH-SY5Y cells were treated with 200 μM or 300 μM of H_2_O_2_ for 24 h, the mRNA levels of IRP2 showed that no significant difference (Figure 2A, *P* > 0.05), whereas the protein levels of IRP2 were decreased in comparison with the 0 μM H_2_O_2_ group (Figure 2B, *P* < 0.01). Thus, H_2_O_2_ regulated IRP2 expression in protein levels. The SH-SY5Y cells were incubated with 100 μM Fe^2+^ to detect the ability of cell iron uptake. Cell iron uptake ability was observed using laser confocal microscopy, and the results showed that the intracellular fluorescence intensity was increased with time, indicating a decreased Fe^2+^ uptake in both 200 μM and 300 μM H_2_O_2_ treatment groups (Figure 2C, *P* < 0.05). Moreover, the expression of TfR1in 200 and 300 μM H_2_O_2_ treatment groups were also decreased (Figure 2D, *P* < 0.05). These results suggested that H_2_O_2_ treatment resulted in a decrease of IRP2 protein levels rather than mRNA levels, leading to down-regulation of IRP2 targets and iron uptake ability.

### The Down-Regulative Effects of H_2_O_2_ on IRP2 Protein Levels Through Ubiquitination Pathway

**FIGURE 3:**
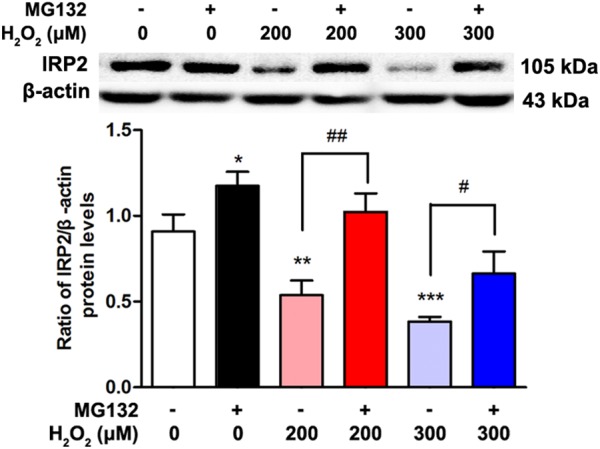
The expression of IRP2 after treated by H_2_O_2_ and MG132. Data were presented as mean ± SEM of 3 independent experiments. One-way ANOVA, ^∗^*P* < 0.05, ^∗∗^*P* < 0.01, and ^∗∗∗^*P* < 0.001 compared with 0 μM H_2_O_2_ group. *T*-test,^#^*P* < 0.05 and ^##^*P* < 0.01.

We next examined how such H_2_O_2_ affected IRP2 protein levels. To determine the expression of IRP2 that induced by H_2_O_2_ through ubiquitination pathway, we incubated SH-SY5Y cells with the ubiquitin proteasome inhibitor MG132. Figure 3 showed that the expression levels of IRP2 levels were decreased in H_2_O_2_ treatment groups and were restored by MG132 treatment, the difference was statistically significant (*P* < 0.05).

### FBXL5 Was Involved in the Degradation of IRP2 Induced by H_2_O_2_

**FIGURE 4:**
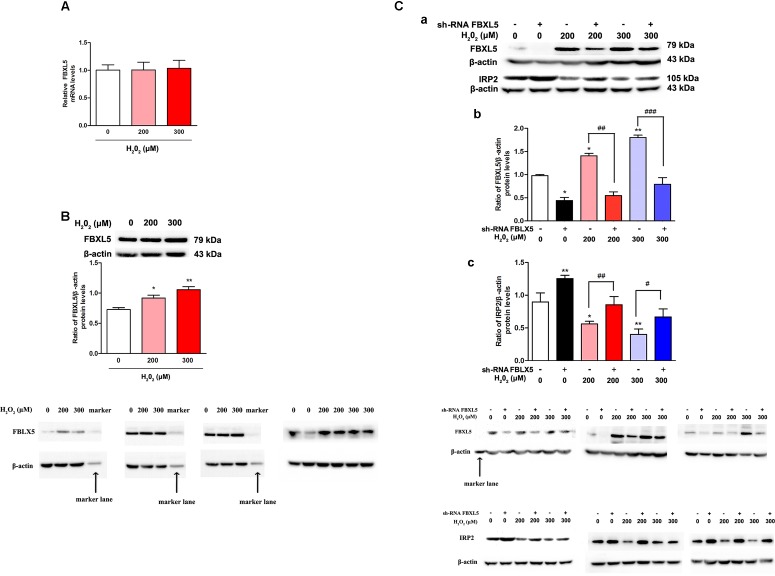
FBXL5 was involved in the degradation of IRP2 induced by H_2_O_2_ treatment in SH-SY5Y cells. **(A,B)** FBXL5 mRNA **(A)** and protein **(B)** expression were observed in SH-SY5Y cells treated by H_2_O_2_. One-way ANOVA, data were presented as mean ± SEM of 3 independent experiments. ^∗^*P* < 0.05 and ^∗∗^*P* < 0.01 compared with 0 μM H_2_O_2_ group. Data were presented as mean ± SEM of 3 independent experiments. One-way ANOVA, ^∗^*P* < 0.05 and ^∗∗^*P* < 0.01 compared with 0 μM H_2_O_2_ group. *T*-test,^#^*P* < 0.05 and ^##^*P* < 0.01. **(C)** Pretreatment with si-FBXL5, the expression of FBXL5 and IRP2 changed after treated by H_2_O_2_ and si-FBXL5. Data were presented as mean ± SEM of 3 independent experiments. One-way ANOVA, ^∗^*P* < 0.05 and ^∗∗^*P* < 0.01 compared with H_2_O_2_ 0 μM group. *T*-test,^#^*P* < 0.05, ^##^*P* < 0.01, and ^###^*P* < 0.001.

F box and leucine-rich repeat protein 5, an E3 ubiquitin ligase subunit, is a key event to mediate IRP2 degradation for controlling iron homeostasis (Moroishi et al., 2011). To test our hypothesis that IRP2 reduction was raised by FBXL5 in the oxidative environment, we measured the FBXL5 expression both in mRNA and in protein levels. Results showed that the mRNA levels of FBXL5 showed no significant difference (Figure 4A, *P* > 0.05), whereas the protein levels of FBXL5 were increased in both H_2_O_2_ treatment groups (Figure 4B, *P* < 0.05). These data indicated that oxidative factors H_2_O_2_ could cause the decrease of IRP2 and the increase of FBXL5.

Sh-FBXL5 were transfected into SH-SY5Y cells, and we found that FBXL5 levels were decreased in all H_2_O_2_ treatment groups (Figure 4Ca,b, *P* < 0.05), and IRP2 levels were increased in all groups (Figure 4Ca,c, *P* < 0.05), the difference was statistically significant in comparing with negative control. The results suggested that H_2_O_2_ could down regulate IRP2 through FBXL5 mediated ubiquitination pathway.

## Discussion

We have shown that the oxidative stress induced by H_2_O_2_ in SH-SY5Y cells resulted in a down-regulation of IRP2 protein levels, which in turn leads to a decrease of iron uptake ability. The IRP2 protein stability was regulated by ubiquitination pathway. FBXL5, the E3 of IRP2, was up-regulated after treatment of H_2_O_2_. The knockdown of FBXL5 could attenuate the IRP2 down-regulation that induced by H_2_O_2_. Our findings indicate that H_2_O_2_ regulated IRP2 expression through FBXL5 mediated ubiquitination pathway in SH-SY5Y cells.

Molecules or molecular fragments containing one or more unpaired electrons are called free radicals. These free radicals in living systems are mostly derived from oxygen (Jomova et al., 2010). The ROS, such as (O_2_^-^), derived from metabolic processes, and then further interacts with other molecules via enzyme- or metal-catalyzed to generate “secondary” ROS (Jomova et al., 2010; Farina et al., 2013). Excessive exposure to ROS may induce oxidative stress particularly in mitochondria, and lead to neurodegenerative diseases. H_2_O_2_ generates oxygen by catalase, which lead to hyperoxia. And the free intracellular Fe^2+^and H_2_O_2_ participate in the Fenton reaction to generate high reactive hydroxyl radical. Thus, in the present study, we used H_2_O_2_ mimic oxidative stress (Figure 1). The cell viability was reduced with the increased H_2_O_2_ concentrations (Figure 1A). The ΔΨm reduction and excessive generation of ROS increased both in H_2_O_2_ (Figure 1B,C) treatment groups. These results indicated that cells were damaged by the artificial oxidative stress *in vitro*.

In the present study, we found that H_2_O_2_ reduced the expression of IRP2 just in protein levels rather than mRNA levels in the dopaminergic-like neuroblastoma cells (Figure 2A,B). Correspondingly, TfR1 expression and cell iron uptake ability were reduced. As a key regulator of brain iron metabolism, IRP2 expression is accurately modulated by the microenvironment factors, including oxygen, iron, free radicals, and cytokines. Generally, neurodegenerative diseases have high oxidative stress level (Emerit et al., 2004; Kim et al., 2015). For example, oxidative stress is especially substantiated by the complex I mitochondrial dysfunction with increased ROS production that might induced by accumulated α-synuclein and iron in the SN of PD patients (Jomova et al., 2010; Farina et al., 2013; Hwang, 2013). In our pervious study, we found that the expression of IRP2 mRNA levels was not affected by FAC at concentration of 100 μM (Zhang et al., 2009). Moreover, sodium nitroprusside, a NO donor and an oxidative generator, could inhibit the IRP2 protein expression (Wei et al., 2017). The mechanism underlying oxidative stress-mediated down-regulation of IRP2 is complex. IRP2 mRNA transcription is regulated by HIF-α of transcription factors that affected by oxygen and iron (Kaelin and Ratcliffe, 2008). In post-transcriptional regulation, IRP2 was recognized by ubiquitin ligase and then degraded by the proteasome in kidney cell line (Salahudeen et al., 2009; Takahashi-Makise et al., 2009; Vashisht et al., 2009). However, the mechanisms of oxidative stress affect IRP2 expression in neurodegenerative diseases are largely unknown. We found that MG132 treatment could prevent the reduction of IRP2 that induced by H_2_O_2_ (Figure 3), suggesting that oxidative stress regulated IRP2 expression in dopaminergic-like neuroblastoma cells might be achieved through ubiquitination pathway.

There are two E3 ubiquitin ligases for IRP2, including FBXL5 and haem-oxidized IRP2 ubiquitin ligase 1 (HOIL-1). HOIL-1 belongs to RING finger protein and recognizes IRP2 through a signal created by heme-mediated oxidative modification of the protein (Iwai et al., 2005). However, HOIL-1 is independent and is not required for iron-dependent degradation of IRP2 (Zumbrennen et al., 2008). The selective iron- and oxygen-dependent degradation of IRP2 is mediated by the Skp1/Cul1/Fbox (SCF) E3 ubiquitin ligase complex containing FBXL5 (Salahudeen et al., 2009; Vashisht et al., 2009). Disruption of the FBXL5 expression are failed to sense the increased cellular iron availability, which results in constitutional accumulation of IRP2 and the disordered expression of its target genes (Salahudeen et al., 2009; Vashisht et al., 2009). In *FBXL5* conditional deleted mice, hematopoietic stem cells are cellular iron overload and reduced in cell number (Muto et al., 2017). It is also found that *FBXL5*^-/-^ mice die for overwhelming accumulation of oxidative stress during embryogenesis (Moroishi et al., 2011). Here, we found that FBXL5 increased and its targets IRP2 reduced when treated by H_2_O_2_ in SH-SY5Y cells. Thus, FBXL5 might be also referred to regulating IRP2 expression under oxidative stress condition in nerve system.

F box and leucine-rich repeat protein 5 contains a hemerythrin domain in the N-terminus that belongs to a family of iron- and oxygen-binding proteins, and contains the leucine-rich repeats in C-terminal region that binds to IRP2 (Salahudeen et al., 2009; Chollangi et al., 2012; Ruiz and Bruick, 2014). The N-terminal hemerythrin domain has iron and oxygen sensing properties for binding with Fe-O-Fe center. The Fe-O-Fe center is formed in iron replete and oxygenated cells. FBXL5 is stable when directly binds to iron in the hemerythrin domain, whereas unstable under iron-deficient conditions. With this iron-sensing ability, FBXL5 controls the abundance of IRP2 in an iron-dependent manner, and degrades IRP2 by the stabilized FBXL5 under iron-replete conditions. In addition to iron bioavailability, IRP stability and activity are regulated by oxygen. Acting as ROS sensors, the hemerythrin domain could also facilitate regulation by ROS that stabilizes FBXL5 and antagonizes the stabilization of IRP2 (Ruiz and Bruick, 2014). In our present study, oxidative stress generators H_2_O_2_ could up-regulate FBXL5 expression (Figure 4A,B) and using si-FBXL5 could alleviate the reduction of IRP2 protein levels (Figure 4C), suggesting that the close relationship of FBXL5 and oxidative stress in regulation nerve system cellular iron metabolism.

In conclusion, we present novel evidence that the oxidative generators H_2_O_2_ induced an increase of FBXL5-mediated ubiquitination degradation in dopaminergic-like neuroblastoma cells. The results of this study implicate an important role of oxidative stress in regulating iron metabolism in nerve system.

## Author Contributions

HJ and QJ conceived the project and designed the study. QJ, JW, and XD performed the experiments, analyzed the data, and interpreted the results. QJ wrote the manuscript. HJ and YL reviewed and edited the manuscript. All authors have read and approved the final version of the manuscript.

## Conflict of Interest Statement

The authors declare that the research was conducted in the absence of any commercial or financial relationships that could be construed as a potential conflict of interest.
